# Effect of β-hydroxy-β-methylbutyrate on miRNA expression in differentiating equine satellite cells exposed to hydrogen peroxide

**DOI:** 10.1186/s12263-018-0598-2

**Published:** 2018-04-10

**Authors:** Karolina A. Chodkowska, Anna Ciecierska, Kinga Majchrzak, Piotr Ostaszewski, Tomasz Sadkowski

**Affiliations:** Department of Physiological Sciences, Faculty of Veterinary Medicine, Warsaw University of Life Sciences – SGGW, Nowoursynowska 159, 02-776 Warsaw, Poland

**Keywords:** miRNA, HMB, Equine satellite cells, Muscle injury, Skeletal muscle

## Abstract

**Background:**

Skeletal muscle injury activates satellite cells to initiate processes of proliferation, differentiation, and hypertrophy in order to regenerate muscle fibers. The number of microRNAs and their target genes are engaged in satellite cell activation. β-Hydroxy-β-methylbutyrate (HMB) is known to prevent exercise-induced muscle damage. The purpose of this study was to evaluate the effect of HMB on miRNA and relevant target gene expression in differentiating equine satellite cells exposed to H_2_O_2_. We hypothesized that HMB may regulate satellite cell activity, proliferation, and differentiation, hence attenuate the pathological processes induced during an in vitro model of H_2_O_2_-related injury by changing the expression of miRNAs.

**Methods:**

Equine satellite cells (ESC) were isolated from the samples of skeletal muscle collected from young horses. ESC were treated with HMB (24 h) and then exposed to H_2_O_2_ (1 h). For the microRNA and gene expression assessment microarrays, technique was used. Identified miRNAs and genes were validated using real-time qPCR. Cell viability, oxidative stress, and cell damage were measured using colorimetric method and flow cytometry.

**Results:**

Analysis of miRNA and gene profile in differentiating ESC pre-incubated with HMB and then exposed to H_2_O_2_ revealed difference in the expression of 27 miRNAs and 4740 genes, of which 344 were potential target genes for identified miRNAs. Special attention was focused on differentially expressed miRNAs and their target genes involved in processes related to skeletal muscle injury. Western blot analysis showed protein protection in HMB-pre-treated group compared to control. The viability test confirmed that HMB enhanced cell survival after the hydrogen peroxide exposition.

**Conclusions:**

Our results suggest that ESC pre-incubated with HMB and exposed to H_2_O_2_ could affect expression on miRNA levels responsible for skeletal muscle development, cell proliferation and differentiation, and activation of tissue repair after injury. Enrichment analyses for targeted genes revealed that a large group of genes was associated with the regulation of signaling pathways crucial for muscle tissue development, protein metabolism, muscle injury, and regeneration, as well as with oxidative stress response.

**Electronic supplementary material:**

The online version of this article (10.1186/s12263-018-0598-2) contains supplementary material, which is available to authorized users.

## Background

β-Hydroxy-β-methylbutyrate (HMB) is a metabolite of the essential amino acid leucine and is naturally synthesized in animals, plants, and humans [1]. Dietary supplementation of HMB is used to enhance gains in strength and lean body mass associated with resistance training and for increasing lean mass in cancer-related cachexia [2, 3]. Unlike anabolic hormones which only increase muscle protein synthesis to accelerate muscular hypertrophy, HMB increases dynamic strength [4, 5] and lean body mass [6] acting as an anti-catabolic agent, reducing protein breakdown [5] and cellular damage which may accompany intense exercise [7]. Moreover, previous studies have demonstrated that HMB supplementation decreased plasma post-exercise creatine kinase and lactic acid in thoroughbreds [8].

Reactive oxygen species (ROS), such a hydrogen peroxide (H_2_O_2_), exert a critical regulatory role on skeletal muscle function [9, 10]. In resting muscle cells, free radicals and ROS are rapidly and efficiently neutralized by antioxidants. Exercise creates an imbalance between ROS and activates natural antioxidant mechanisms. Moreover, ROS produced during exercise by inflammatory cells may also be involved in delayed onset of muscle damage observed during inflammation [11]. The inflammatory response coincides with muscle repair, regeneration, and growth, involving activation and proliferation of satellite cells followed by their terminal differentiation. In response to the damage, quiescent satellite cells are activated and undergo several cycles of cell division prior to their withdrawal from the cell cycle through terminal differentiation and finally fusion with the damaged skeletal muscle fibers [12]. During training-related tissue microdamage, activation of satellite cells is considered to play a crucial role in injured muscle fibers by incorporating new myonuclei and thus increasing muscle size and strength (by hypertrophy) [13].

MicroRNAs (miRNAs) are small non-coding interfering RNA molecules (18–25 nucleotides) able to post-transcriptionally regulate gene expression through sequence-specific base pairing to messenger ribonucleic acid (mRNA). These molecules have been shown to be important key players in a variety of physiological and pathological processes (proliferation, differentiation, apoptosis, hypertrophy, timing development, inflammation, cancer, etc.). A group of miRNAs, highly enriched in skeletal and/or cardiac muscles (myomiRs), has recently been identified and includes miR-1, miR-133a, miR-133b, miR-206, miR-208, miR-208b, miR-486, and miR-499 [14] which regulate skeletal muscle development.

Szcześniak et al. [15] were the first who demonstrated the effect of HMB in ESC. Our study was performed to evaluate miRNA profile and relevant target genes in differentiating equine satellite cells incubated with HMB and also exposed to H_2_O_2_ an in vitro factor initiating cellular response similar to that observed in vivo during a short intensive physical exercise and post-exercise injury.

## Methods

### Muscle samples and cell culture

Samples of skeletal muscles (*m. semitendinosus*) were collected from 6 months old healthy stallions in a slaughter house. Muscle samples (0.5 × 0.5 × 0.5 cm) were taken immediately, washed in phosphate*-*buffered saline (PBS) with gradually decreasing antibiotic concentration [40.000 and 20.000 IU *Penicillium crystalicum* (PC; Polfa, Poland) per 100 ml PBS], cleaned from connective and fat tissue, cut and immediately suspended in sterile fetal bovine serum (FBS; Life Technologies, USA) with 10% addition of dimethylsulfoxide (DMSO), gradually frozen to − 80 °C, and finally stored in liquid nitrogen until use.

### Satellite cell isolation, proliferation, and differentiation

Equine satellite cells (ESC) were isolated according to the following protocol. Protease from *Streptomyces griseus* (Pronase®, Sigma-Aldrich, USA) was reconstituted in low-glucose Dulbecco's modified Eagle’s medium (DMEM), GlutaMAX™, Pyruvate (Life Technologies, USA), and stirred for 1 h, pH 7.3. The incubation buffer (IB) consisted per sample of Pronase 0.5 mg/ml, 18 ml of DMEM, FBS 2 ml (Life Technologies, USA), and PC (20.000 IU). IB was filtered through a cellulose acetate membrane syringe filter (Sigma-Aldrich, USA). The fragmented muscle tissue was thawed, washed in PBS with PC (20.000 IU), and suspended in IB for 1.5 h at 37 °C, shaken every 15 min. Then, samples were sieved through cell strainer (70 μm, nylon, Falcon, USA)*.* The filtrate was centrifuged for 20 min (350 g), which was repeated three times. After each centrifugation, supernatant was discarded, cell pellet was re-suspended in growth medium (GM; 10%FBS/10% horse serum (HS) in DMEM (Life Technologies, USA) and antibiotics (AB; 0.5% amphotericin B (Fungizone, Life Technologies, USA), 1% penicillin-streptomycin (Life Technologies, USA)). After the last centrifugation, cell suspension was transferred to polystyrene Petri dishes (Becton Dickinson, USA) for 1.5 h to allow adhesion of fibroblast. After that, supernatant with satellite cells was transferred into culture dishes (Primaria Cell Culture Flask, Becton Dickinson, USA) and cultured in GM. The growth medium was changed every 2 days. On the tenth day of proliferation, cells were trypsinized, counted by Scepter Cell Counter (Merck Millipore, Germany), transferred (30,000 cells from each isolation) to Collagen I Cellware six-well plate (Greiner Bio-One, USA), and cultured in GM. After reaching 80% confluency, the proliferation media was replaced by the differentiation media (DM; 2%HS in DMEM with AB).

**Fig. 1:**
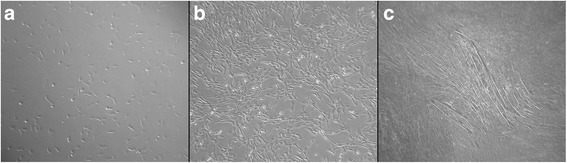
Equine satellite cell culture. **a** Proliferating ESC, day 4. **b** Proliferating ESC, day 8. **c** Myotubes in differentiating ESC, day 2

Primary satellite cell cultures from semitendinosus muscle of all horses were isolated, and the culture with the best scores of cell viability (MTT assay) [16] and fusion index was selected for further analysis (data not shown). Different stages of equine satellite cell culture are presented in Fig. 1.

### Experimental design

**Fig. 2:**
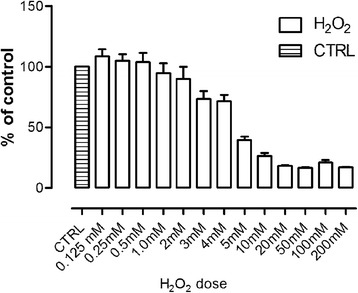
H_2_O_2_ dose-dependent effect on ESC cell viability assessed by MTT assay

**Fig. 3:**
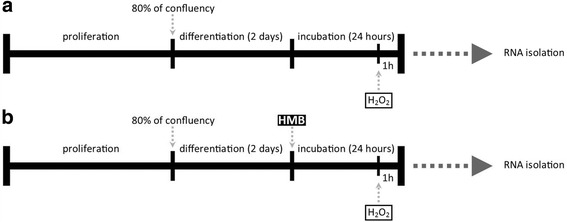
Experimental design. **a** Control group. **b** HMB-treated group

After the second day of differentiation, 50 μM HMB (Metabolic Technologies Inc., USA) was added to the culture media, and then, cells were incubated for an additional 24 h. Ca-HMB was purchased from MTI (USA). The free HMB acid was extracted by acidification and organic extraction [8]. HMB dose was chosen based upon previous studies [3, 17] and MTT assay results which confirmed literature data (data not shown). During the last hour of incubation, 3 mM hydrogen peroxide (solution 30% (*w*/*w*) in H_2_O (Sigma-Aldrich, USA) was added to induce cell damage. Due to the lack of literature data on the doses of H_2_O_2_ used in the equine satellite cell culture, the MTT assay was performed using doses ranging for 0.125 to 50 mM. Compared to the previously described doses of H_2_O_2_ used in other cell culture models, those used for ESCs were relatively large. For this reason, we decided to use H_2_O_2_ dose 3 mM with DL-25 (Fig. 2). The experimental design is presented in Fig. 3.

### RNA isolation

After the H_2_O_2_ treatment, the differentiating ESCs were scraped and total RNA was isolated using a miRNeasy Mini Kit (Qiagen, USA) according to the manufacturer’s protocol. The quantity of RNA was measured spectrophotometrically using NanoDrop 2000 (Thermo Scientific, USA). The quality of the total RNA was verified by Bioanalyzer 2100 (Agilent, USA), and only samples with RIN ≥ 9.2 were used for further analysis.

### Microarray analysis

For the microRNA profiling, the Custom Equine miRNA 8x15K Microarray slides were designed using eArray platform (https://earray.chem.agilent.com/earray, GEO database: GPL20990) and provided by Agilent Technologies (USA).

MiRNA was isolated from eight equine satellite cell cultures for both HMB pre-treated (*n* = 8) and control group (*n* = 8). As recommended by Agilent Technologies (USA), 100 ng of total RNA of each sample was taken and labeled using miRNA Complete Labeling and Hyb Kit (version 2.3, December 2010). For hybridization, Microarray Hybridization Chamber (Agilent, USA) and Hyb-Buffer (Agilent, USA) were used according to the manufacturer’s protocol. In the next step, slides were washed out using Gene Expression Wash Pack (Agilent, USA) and scanned in Microarray Scanner (model G2565CA) with SureScan High-Resolution Technology (Agilent, USA).

Microarray data were extracted, the background was subtracted, and normalization was performed using the standard procedures included in the Agilent Feature Extraction (FE) Software version 10.7.3.1.

Analysis of gene expression (GE) profile was performed using Horse Gene Expression Microarray, 4x44K (Agilent Technologies, USA) based on the same protocol as described by Szcześniak et al. (2016), [15, 18]. Briefly, two-color microarray, with 825 ng of cRNA from HMB-exposed cells (labeled by Cy5, *n* = 4) and 825 ng of cRNA from control cells (labeled by Cy3, *n* = 4), and RNA Spike-In Kit (Agilent Technologies, USA) as an internal control were used. The background was subtracted and Linear and Lowess normalization was performed using the standard procedures included in the Agilent Feature Extraction (FE) Software version 10.7.3.1. The data were statistically analyzed using Gene Spring 13.0 software (Agilent, USA). The statistical significance of the differences was evaluated using Student’s *t* test (*p* < 0.05) and Benjamini and Hochberg multiple testing correction. False Discovery Rate (FDR) ≤ 0.05 and fold change (FC) ≥ 1.3 were considered as statistically significant. Microarray data were deposited at the Gene Expression Omnibus data repository under the number GSE73779 for miRNA and GSE93025 for cDNA.

### Real-time qPCR

The criteria for miRNA and differentially expressed gene (DEG) selection for real-time qPCR validation and further analysis were of biological relevance (miRNAs linked to muscular development, hypertrophy, muscle injuries, oxidative stress, and tissue regeneration) and were assessed based on Pathway Studio Mammalian (Elsevier, USA) and available literature.

For miRNA, real-time qPCR validation miRCURY LNA™ Universal RT microRNA PCR kit (Exiqon, USA) was used. A two-step protocol was applied: (1) polymerase activation at 95 °C for 10 min and (2) 40 amplification cycles at 95 °C for 10s and 60 °C for 1 min, according to the manufacturer protocol.

Primers were chosen based on the miRNA sequences assigned to microarray probes and were provided by Exiqon (Denmark) (Table 1). Calculation of the relative miRNA expression using the ΔΔCt method was applied using GenEX 6 software provided by MultiD (Sweden). Obtained data were statistically analyzed using two-tailed Student’s *t* test. Values of *p* ≤ 0.05 were considered statistically significant.

**Table 1:** Primers for real-time qPCR: primers for miRNA

No.	Primer’s name	Target sequence	Amplification time and temperature
1	miR-146a-5p	UGAGAACUGAAUUCCAUGGGUU	10 s/95 °C and 60 s/60 °C
2	miR-146b-5p	UGAGAACUGAAUUCCAUAGGCU	10 s/95 °C and 60 s/60 °C
3	miR-204b	UUCCCUUUGUCAUCCUAUGCCU	10 s/95 °C and 60 s/60 °C
4	miR-208b	AUAAGACGAACAAAAGGUUUGU	10 s/95 °C and 60 s/60 °C
5	miR-222	AGCUACAUCUGGCUACUGGGU	10 s/95 °C and 60 s/60 °C
6	miR-675	UGGUGCGGAGAGGGCCCACAGUG	10 s/95 °C and 60 s/60 °C

Based on previous studies in different species and the manufacturer recommendation (Exiqon, Denmark), a U6 snRNA reference was used. To verify GE microarray results, the real-time qPCR method was applied. All the steps of real-time qPCR procedure were made based on the protocols previously described by Szcześniak et al. (2016), [15]. The sequences of primers are listed in Table 2. *Gapdh* was used as a reference gene.

**Table 2:** Primers for real-time qPCR: primers for mRNA

No.	Primer’s name	Forward sequence	Reverse sequence	Annealing time and temperature
1	*otud4*	CCACCTCCTGCAGAACAAAAG	GGGTCTTGGTAAAGGCTCATT	15 s/60 °C
2	*cx43* (*gja1*)	TGCTGCGAACCTACATCATC	CGATGACGTTCAAGGCAAGA	15 s/60 °C
3	*sod2*	GTCACCGAGGAGAAGTACCA	CTGGTTAGGACAGGCAACAA	15 s/60 °C
4	*sod1*	GATTCCACGTCCACGAGTTT	CCCGAGAGTGAGATCACAGA	15 s/60 °C
5	*tgfb2*	AGTACTACGCCAAGGAGGTT	TAGGCGGGATGGCATTTTCC	15 s/60 °C
6	*myf5*	GGAGACGCCTGAAGAAAGTC	CCGGCAGGCTGTAGTAATTC	15 s/60 °C
7	*bdnf*	CCCCATGAAAGAAGCAAACG	TACAAGTCCGCGTCCTTACT	15 s/60 °C
8	*gapdh*	GTTTGTGATGGGCGTGAACC	GTCTTCTGGGTGGCAGTGAT	15 s/60 °C

### Target gene prediction and ontological analyses

MicroRNA target gene prediction was performed using the TargetScan database. The analysis was performed for all identified HMB-affected miRNAs. For each predicted target of individual miRNA, the sum of the context + scores was automatically calculated. Predicted targets of each miRNA family were automatically sorted by total context + score. Analysis was performed for the context score percentile (50) and conserved/non-conserved miRNA families and target sites [19]. For further analysis, common genes for those identified genes using GE microarray and predicted miRNA target genes were selected and considered as targets for HMB treatment-influenced miRNAs.

Ontological analyses revealing molecular functions, biological processes, and pathways of miRNA targets were performed in DAVID 6.7 using Fisher’s exact test with *p* ≤ 0.05. Detailed analysis of the role of HMB-modulated miRNAs, genes identified using GE, and target genes in various metabolic and signal pathways was performed using Pathway Studio Web (Elsevier, USA). Relationships between all differentially expressed miRNAs were visualized with Pathway Studio’s Build Pathway functionality which is based on the wave-propagation algorithm developed for the navigation through complex networks. Find Direct Links/All objects Directions Algorithm was used in this analysis.

### Western blot analysis

The procedure of Western blot analysis was performed based on the previously described methodology by Zielniok et al. [20]. Antibodies used in Western blot were against the following: SOD1 (ab62800), SOD2 (ab13534), TGFβ2 (sc-90), α-tubulin (ab176560), BDNF (sc-546), MYF5 (sc-302), GAPDH (sc-20357), and β-actin (sc-47778).

### Cell viability, cell damage, and oxidative stress

Hydrogen peroxide, used in the experiment as a damage factor, is known to affect various cellular processes. Several tests related to the cell viability, cell damage, and oxidative stress were performed to assess the impact of HMB on the cellular processes following incubation with H_2_O_2_. Experimental conditions (incubation time, doses of HMB and H_2_O_2_) were the same as previously in the part related to the microarray and real-time qPCR analysis.

CellROX® Green Reagent Kit (Life Technologies) was used to measure oxidative stress and cell death in ESC’s based on the manufacturer protocol. Cells were seeded on 24-well plates at 0.05 × 10^6^ cells/cm^2^. Cells were incubated for 60 min with CellROX reagent in a final concentration 250 μM. During the last 15 min of staining, SYTOX Red Dead Cell was added (at the final concentration 5 nM). The samples were analyzed immediately after staining using FACS Aria II (BD Biosciences) flow cytometer. A total of 50,000 events per sample (*n* = 3) were collected. This staining was performed on live cells during the proliferative phase (90% confluency). Data were analyzed using FlowJo (TreeStar, USA) and GraphPad Prism software.

The second test related to oxidative stress called Total Antioxidant Capacity (TAC) Assay Kit (Abcam, UK) was used according to the manufacturer protocol. This test can measure either the combination of both small molecule antioxidants and proteins or small molecules alone in the presence of our proprietary Protein Mask. Cells were seeded on 96-well plates at 2 × 10^6^ cells (*n* = 6). After a 90-min incubation, the plate was read on Tekan System reader at 570 nm wavelength. Data were analyzed using GraphPad Prism software.

Lipid peroxidation is the degradation of lipids that may accompany the activity of several cell damage factors including hydrogen peroxide. It is also one of the popular markers for oxidative stress. Lipid peroxidation Assay Kit (Sigma-Aldrich) was used to measure lipid peroxidation. All the procedure was performed based on the provided manufacturer protocol. The concentration of MDA was measured for *n* = 6. The staining was performed on live cells during differentiation phase.

In order to increase the reliability of the obtained results related to cell survival, the MTT test was also performed (*n* = 6) based on the previously published protocol [16]. Data for both tests were analyzed using GraphPad Prism software.

Qualitative flow cytometry assay for mitochondrial depolarization was also performed according to the manufacturer protocol. The 5,5′,6,6′-tetrachloro-1,1′,3,3′-tetraethylbenzimidazolylcarbocyanine iodide (JC-1, Sigma-Aldrich) was used. It is a cationic, lipophilic dye that accumulates in mitochondria and exhibits green fluorescence (525 nm) in its monomeric state. The mostly implemented application of JC-1 is detection of mitochondrial depolarization occurring in the early stages of apoptosis. JC-1 was dissolved in DMSO and medium II for a final concentration of 0.6 μM. The cells were incubated at 37 °C, washed, trypsinized, and resuspended in 2%FBS/PBS medium. Fifty thousand events were collected for each sample using FACS Aria II (BD Biosciences) flow cytometer. Fluorescence compensation was done for 525 nm. This staining was performed on live cells during the proliferative phase (90% of confluency; *n* = 3). Data were analyzed using FlowJo (TreeStar, USA) and GraphPad Prism software.

## Results

In the “Results” and “Discussion” sections, gene symbols are marked in italics and lowercase. The arrows indicate the direction of expression change: ↓ and ↑ for down- and upregulation, respectively.

### Microarray analysis

Analysis of the miRNA expression in differentiating equine satellite cells incubated with HMB (24 h) and exposed to H_2_O_2_ (1 h) revealed differences in 27 miRNAs. Among them, eight demonstrated higher expression and 19 lower expression when compared to control (Table 3).

**Table 3:** MiRNAs differentially expressed in HMB-incubated equine satellite cells exposed to H_2_O_2_, compared to control

No.	Name	Regulation	FC ([HMB] vs [CTRL])	Log FC ([HMB] vs [CTRL])	FDR	miRBase accession no.
1	eca-miR-146a	Up	120.92	6.92	2.74E−18	MIMAT0013065
2	eca-miR-146b-5p	Up	2.68	1.42	9.66E−07	MIMAT0012891
3	eca-miR-204b	Up	1.74	0.79	3.26E−03	MIMAT0013112
4	eca-miR-222	Up	1.60	0.68	7.79E−04	MIMAT0013204
5	eca-miR-155	Up	1.49	0.57	2.30E−03	MIMAT0013182
6	eca-miR-193a-3p	Up	1.39	0.47	9.29E−06	MIMAT0013026
7	eca-miR-221	Up	1.38	0.46	2.11E−03	MIMAT0013203
8	eca-miR-101	Up	1.34	0.42	2.10E−05	MIMAT0012951
9	eca-miR-142-3p	Down	105.23	− 6.72	2.81E−16	MIMAT0013023
10	eca-miR-675	Down	97.37	− 6.60	1.71E−17	MIMAT0013053
11	eca-miR-486-5p	Down	2.21	− 1.14	2.95E−10	MIMAT0013186
12	eca-miR-542-5p	Down	2.10	− 1.07	1.71E−03	MIMAT0013237
13	eca-miR-149	Down	1.86	− 0.89	4.68E−10	MIMAT0012973
14	eca-miR-206	Down	1.66	− 0.73	1.76E−06	MIMAT0013098
15	eca-miR-208b	Down	1.66	− 0.74	1.50E−08	MIMAT0012900
16	eca-miR-133a	Down	1.62	− 0.70	4.99E−09	MIMAT0012997
17	eca-miR-133b	Down	1.59	− 0.67	8.26E−09	MIMAT0013097
18	eca-miR-128	Down	1.58	− 0.66	1.19E−04	MIMAT0013076
19	eca-miR-542-3p	Down	1.53	− 0.61	5.92E−06	MIMAT0013238
20	eca-miR-1	Down	1.49	− 0.57	2.86E−05	MIMAT0012994
21	eca-miR-324-5p	Down	1.49	− 0.58	3.75E−07	MIMAT0013033
22	eca-miR-450c	Down	1.49	− 0.58	2.86E−05	MIMAT0013222
23	eca-miR-450a	Down	1.44	− 0.53	1.19E−05	MIMAT0013219
24	eca-miR-532-3p	Down	1.44	− 0.53	1.02E−07	MIMAT0013236
25	eca-miR-331	Down	1.37	− 0.46	1.60E−07	MIMAT0013188
26	eca-miR-374b	Down	1.34	− 0.42	2.81E−02	MIMAT0013214
27	eca-miR-30c	Down	1.31	− 0.39	2.41E−07	MIMAT0012915

FDR ≤ 0.05, FC ≥ 1.3, *n* = 8

Analysis of gene expression profile for the same experimental conditions as those mentioned above revealed difference in the expression of 4740 transcripts. After removing all duplicate values and unknown sequences, 1923 unique genes were found (Additional file 1: Table S1).

### Functional analysis of identified miRNAs and differentially expressed genes (DEG)

**Fig. 4:**
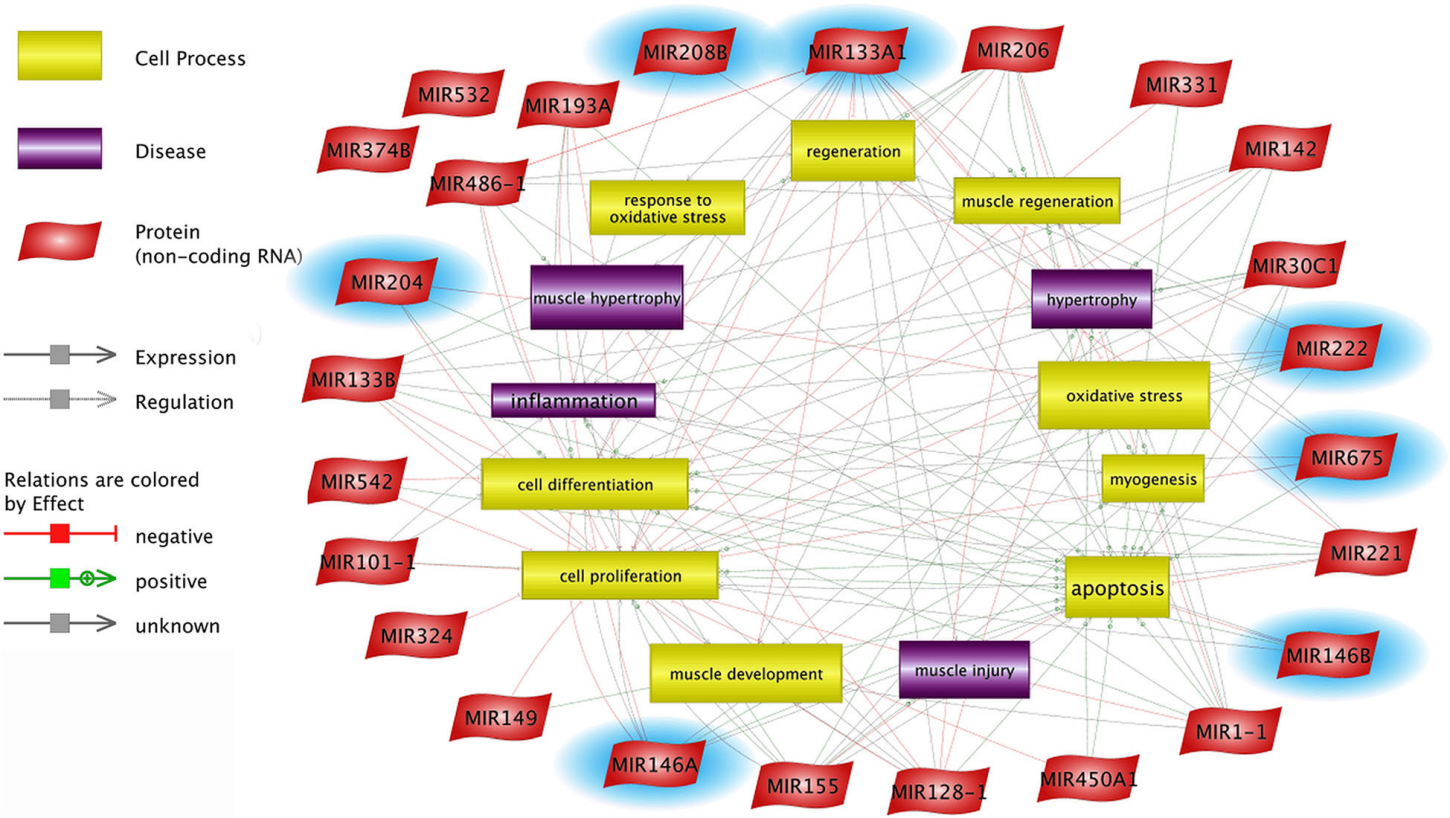
Identified miRNAs and their involvement in some selected physiological and pathological processes. MiRNAs in blue clouds were RT-qPCR-validated

Based upon the Pathway Studio Web Software (Elsevier, USA) and available literature, the results were divided into groups containing miRNAs related to the specific cellular processes, as follows: (1) cell proliferation and differentiation (miR-1, miR-133a/b, miR-206, miR-128, miR-146a/b, miR-204, miR-155, miR-193a, miR-221/222, miR-324, miR-331, miR-374b, miR-486, miR-675), (2) muscle regeneration and hypertrophy (miR-1, miR-133a/b, miR-142, miR-128, miR-146b, miR-208b, miR-675), (3) oxidative stress and inflammation (miR-146a/b), and (4) others (miR-149, miR-30c, miR-532-3p, miR-532-5p, miR-542) (Fig. 4).

Functional analysis showed that GE microarray identified genes were significantly associated with the following biological processes: cellular processes, muscle organ development, proteolysis involved in cellular protein catabolic process, muscle cell differentiation, positive regulation of biological processes, cell death, apoptosis, regulation of cell proliferation, and positive regulation of inflammatory process (Additional file 2: Table S2).

Among identified genes (DEG), special attention has been focused on a few important groups which are known to be HMB affected: muscle organ development (e.g., *six1*, *myf5*, *acta1*, *cav1*, *myh3*, *myh7*, *myl2*, *myl3*, *sgcd*, *tgfb2*), response to wounding/injury (e.g., *jak2*, *igf2*, several members of *cxcl* and interleukin genes, *sod1*, *sod2*), inflammatory response/innate immune response/oxidative stress (*tlr3*, *tlr4*, *tlr10*, *cd40*, *cd44*, *igf2*, *itgb6*, *il-5*, *il-6*, *il-15*, *il-23*, *sod1*, *sod2*, and a large group of chemokine ligand: *ccl*-*1*, *ccl*-*2*, *ccl*-*5*, *ccl*-*8*).

### RT-qPCR validation

**Fig. 5:**
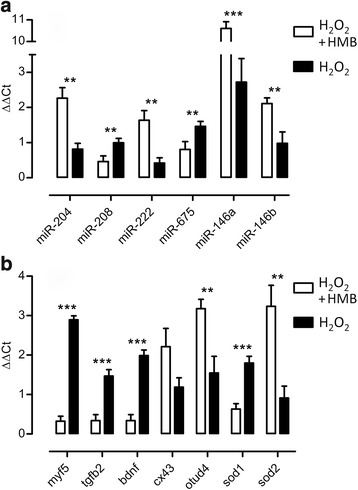
Expression of selected miRNAs (**a**) and genes (**b**) validated by RT-qPCR. Presented values are means ± SE (*p ≤ 0.05; **p ≤ 0.01; ***p ≤ 0.001). HMB—cells treated with HMB and exposed to H_2_O_2_. CTRL—cells without HMB treatment, exposed to H_2_O_2_ (*n* = 6)

From the microarray results, six miRNAs and six genes were selected as a single representative for the aforementioned processes for further RT-qPCR validation. The analysis confirmed statistically significant differences in the expression of six miRNAs (miR-204, miR-208b, miR-222, miR-675, miR-146a, and miR-146b) and six genes (*sod1*, *sod2*, *tgfb2*, *myf5*, *bdnf*, *otud4*) in HMB-treated ESC when compared to control condition (CTRL) (Fig. 5). All RT-qPCR validated miRNAs and genes presented the same trend as microarray results.

### Prediction and ontological analysis of miRNA target genes (DET)

TargetScan analysis was performed to predict potential target genes for all identified miRNAs. The analysis revealed unique 3310 targets for downregulated and 2117 unique targets for upregulated miRNAs. We compared all identified HMB-regulated DEG and aforementioned predicted miRNA target genes to find those which could be regulated by HMB-induced miRNAs in ESC cultures exposed to H_2_O_2_. Finally, 344 differentially expressed target genes (DET) were identified.

Functional analysis showed that DET were associated significantly with several processes which plays an important role in the physiological (protein metabolism, muscle tissue development, cellular homeostasis, apoptosis) and pathological (inflammation, cancer) conditions in muscular tissue (Table 4).

**Table 4:** Selected biological processes in which identified differentially expressed target genes (DET) were involved

Category	Term	Count	FDR
GOTERM_BP_DIRECT	Skeletal muscle tissue development	5	5.4E0
GOTERM_BP_DIRECT	Regulation of inflammatory response	5	7.6E0
GOTERM_BP_DIRECT	Positive regulation of protein catabolic process	5	1.7E1
GOTERM_BP_DIRECT	Actin filament organization	5	2.5E1
GOTERM_BP_DIRECT	Positive regulation of Notch signaling pathway	4	3.6E1
GOTERM_BP_DIRECT	Protein ubiquitination involved in ubiquitin-dependent protein catabolic process	7	3.7E1
GOTERM_BP_DIRECT	Muscle cell cellular homeostasis	3	4.5E1
GOTERM_BP_DIRECT	Regulation of apoptotic process	7	5.0E1

Signaling pathway analysis showed that 27 identified miRNAs could affected target genes involved in several important signaling pathways related to the processes previously described as modified by HMB and also some other which HMB was suspected to affect. The most meaningful pathways are the following: MAPK, RIG-I, Toll-like receptor, hypertrophic cardiomyopathy, ubiquitin-mediated proteolysis, Ras, and response to oxidative stress.

### Western blot analysis

**Fig. 6:**
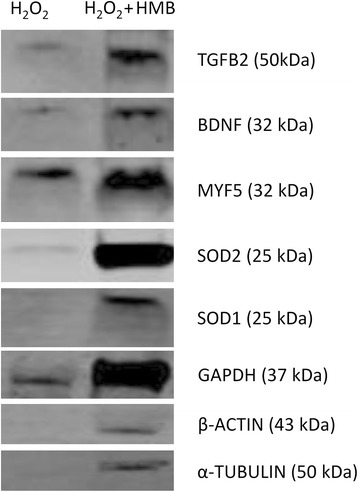
Degradation of protein in H_2_O_2_-treated cell cultures

Western blot analysis of the level of reference proteins and five proteins related to the muscle tissue, muscle damage, and oxidative stress was performed. However, the results are difficult to interpret. Protein degradation at different levels was observed in all samples treated only with hydrogen peroxide (Fig. 6). In groups pre-incubated with HMB and H_2_O_2_, protein degradation was smaller or not observed. It is related to the protein degradation which is strongly linked to hydrogen peroxide effect.

### Cell viability, cell damage, and oxidative stress

**Fig. 7:**
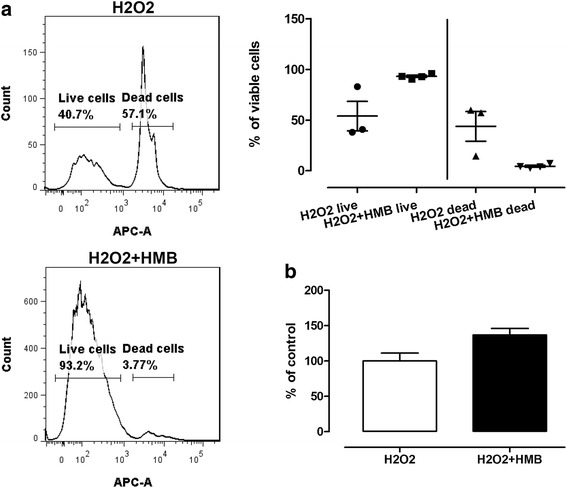
Effect of HMB on cell viability measured by SYTOX Red Cell Dead assay (**a**) and MTT assay (**b**). Each value is the mean ± standard error of the results (*n* = 3, SYTOX Red Cell Dead assay; *n* = 6, MTT assay). Statistical analysis was performed using the one-way ANOVA (*p* < 0.05) and unpaired *t* test (*p* < 0.05) for SYTOX Red Cell Dead and MTT assay, respectively. H_2_O_2_—cells without HMB treatment and exposed to H_2_O_2_. H_2_O_2_ + HMB—cells treated with HMB and exposed to H_2_O_2_

To measure cell viability, two tests were used—MTT and SYTOX Red Dead Cell (as a component of CellROX Green Reagent kit). In both tests, increased cell viability and decreased amount of dead cells were observed in a group pre-treated with HMB and incubated with H_2_O_2_ than in a control group (incubated only with H_2_O_2_). All the results from these two tests were statistically significant (*p* < 0.05). The results of SYTOX Red Dead Cell (A) and MTT test (B) are presented in Fig. 7.

**Fig. 8:**
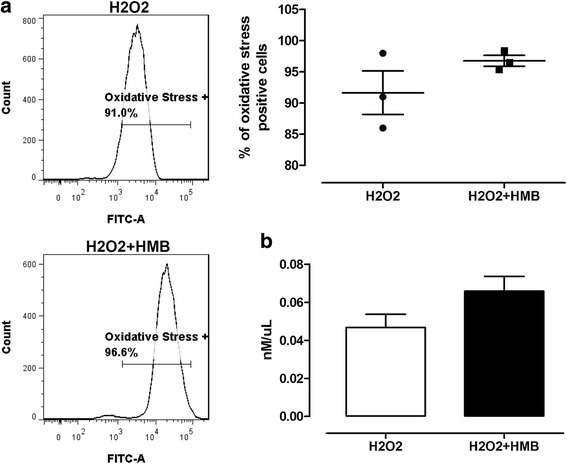
Effect of HMB on oxidative stress—CellROX® Green assay (**a**) and Lipid peroxidation assay (**b**). Each value is the mean ± standard error of the results (*n* = 3, CellROX® Green assay; *n* = 6, lipid peroxidation assay). Statistical analysis was performed using the one-way ANOVA (*p* < 0.05) and unpaired *t* test (*p* < 0.05) for CellROX® Green assay and lipid peroxidation assay, respectively. H2O2—cells without HMB treatment and exposed to H_2_O_2_. H_2_O_2_ + HMB—cells treated with HMB and exposed to H_2_O_2_

Oxidative stress was measured using CellROX® Green Reagent. There was no significant difference between groups (Fig. 8a). Similar results were obtained with the test for lipid peroxidation. There were no statistically significant differences between HMB pre-treated group and control. However, surprisingly, higher lipid peroxidation trend was observed in a HMB pre-treated group compared to control (Fig. 8b).

**Fig. 9:**
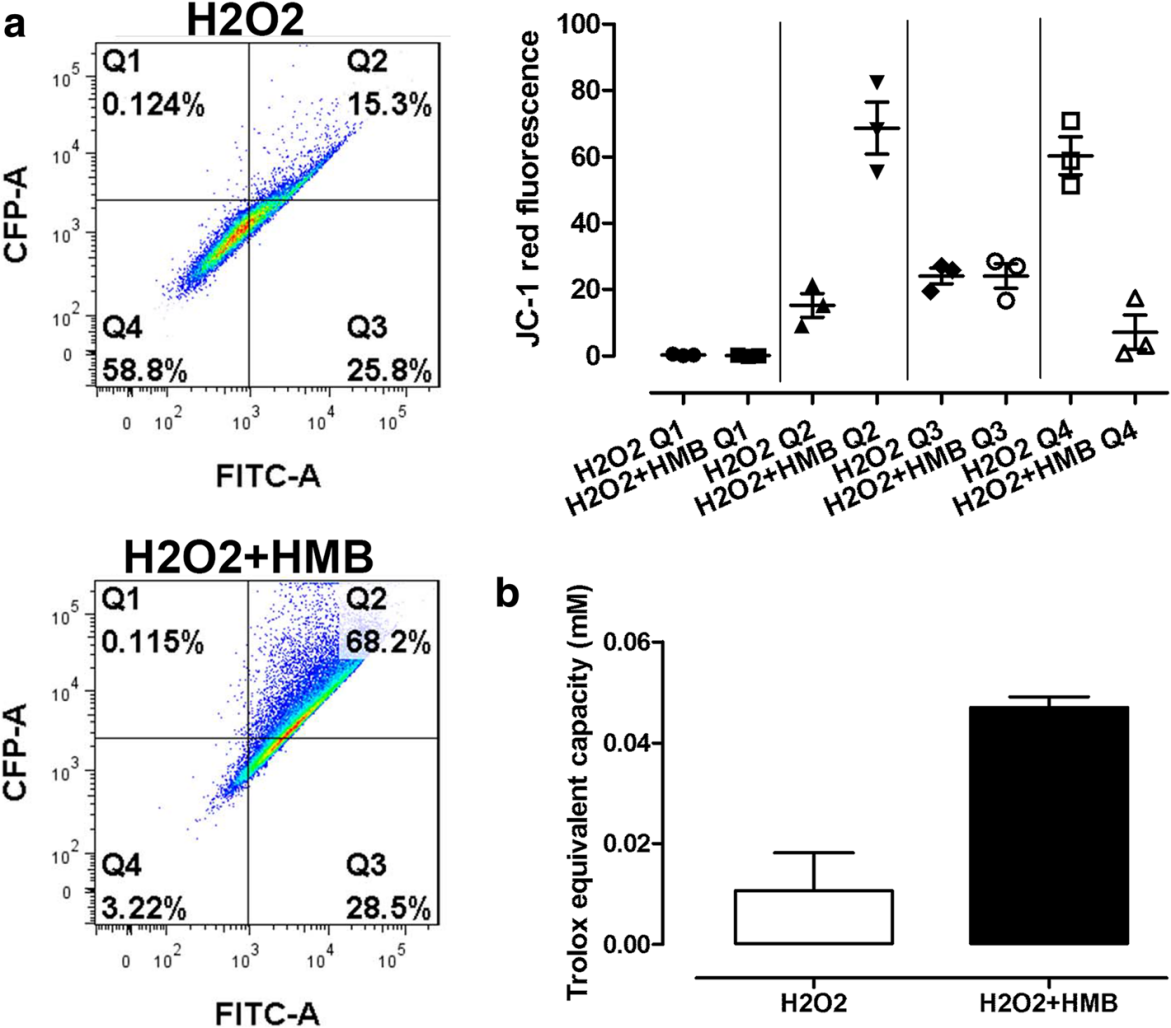
Qualitative flow cytometry assay for mitochondrial depolarization (**a**) and total antioxidant capacity (TAC) (**b**). Each value is the mean ± standard error of the results (*n* = 3, JC-1 assay; *n* = 6, TAC assay). Statistical analysis was performed using the one-way ANOVA (*p* < 0.05) and unpaired *t* test (*p* < 0.05) for JC-1 assay and TAC assay, respectively. H_2_O_2_—cells without HMB treatment and exposed to H_2_O_2_. H_2_O_2_ + HMB—cells treated with HMB and exposed to H_2_O_2_

Qualitative flow cytometry assay for mitochondrial depolarization (JC-1) showed significant differences between the Q2 population (monomers + aggregates in %) and Q4 population (JC-1) in control and HMB pre-treated group. There was no significant difference between Q1 population (% of aggregates) and Q3 population (% of monomers) (Fig. 9a).

Results obtained in a total antioxidant capacity (TAC) assay showed significant differences between HMB pre-treated and control group. Higher antioxidant capacity was observed in HMB pre-treated group (Fig. 9b).

## Discussion

MicroRNAs are essential regulators for numerous biological processes by modulating gene expression at the post-transcriptional level. Several muscle-specific miRNAs (myomiRs) have been shown to play an important role in normal myoblast proliferation, differentiation, and muscle remodeling in response to different type of factors. Recent studies have begun to link miRNAs and certain muscle-related diseases [21]. Modulation of miRNAs by dietary factors and miRNA-based gene therapies seems to be a promising option for the treatment of cardiac and skeletal muscle diseases [22]. Among dietary additives, HMB seems to be an interesting potential myoprotectant for horses [8]. Previous studies suggest that HMB may be involved in the regeneration processes of skeletal muscles [23]. Moreover, HMB stimulates skeletal muscle satellite cell activation and may potentially increase skeletal muscle regenerative capacity after damage induction [24].

Our objective was to determine the influence of HMB on miRNA and gene expression in differentiating equine satellite cells subjected to damaging activity of hydrogen peroxide, as an in vitro model of short extreme effort-related muscle damage observed in racing and sport horses.

Microarray analysis of total RNA in differentiating ESC incubated with HMB (24 h) and treated with H_2_O_2_ (1 h) revealed the difference in the expression of 27 miRNAs (Table 3) and 4740 DEG (Additional file 1: Table S1) from which 344 DET were chosen (Table 4). Identified miRNAs and a large group of identified genes were previously described as these involved in the pathological and physiological processes in skeletal muscles as well as in other tissues. Selected miRNAs (miR-204 (↑), miR-208b (↓), miR-222 (↑), miR-675 (↓), miR-146a (↑), miR-146b (↑)) and genes (*bdnf* (↓), *sod1* (↓), *sod2* (↑), *tgfb2* (↓), *myf5* (↓), *otud4* (↑)) were validated by RT-qPCR showing the same trend as in microarray analysis.

### HMB effects on miRNAs related to satellite/muscle cell proliferation and differentiation

Of the 27 identified miRNAs, 9 are related to cell proliferation and 13 to differentiation in muscle tissue (Fig. 4). Some of miRNAs seem to be particularly interesting in the context of previous publications confirming proven and potential HMB effect on muscle. Among them, family of miR-146a/b able to balance the induction of muscle proliferation or differentiation with miR-146 up- and downregulation, respectively [25]. The miR-146a was one of the highest differentially expressed molecules showing 120.92 fold change in HMB-treated cells. It could suggest their possible involvement in promotion of HMB-induced myoblast proliferation. It is well-known that activation and proliferation of satellite cells is a prerequisite of skeletal muscle injury repair [12], and it is possible that HMB is capable to influence miRNA expression, increasing myoblast proliferation rate and thus facilitating the myofiber regeneration. Similar observations were done for miR-133, in which upregulation was described as proliferation-inducing while its downregulation was responsible for differentiation progression [26]. Interestingly, miRNA-222/221 which over-expression was noticed in myoblasts undergoing differentiation with its downregulation after differentiation [27] was downregulated in ESC cultures exposed to H_2_O_2_ and pretreated with HMB, when compared to control. The same expression trend (↓) was observed in miR-374b which over-expression is known to impair C2C12 cell differentiation, while inhibition promoted this process [28]. Moreover, three miRNAs (miR-675, miR-324, and miR-331) known to be over-expressed in muscle cell differentiation [29, 30] were downregulated in our experiment. Two other miRNAs, miR-206 and miR-1, known to be downregulated in muscle cell proliferation and upregulated during differentiation [31], have manifested downregulation in ESC cultures treated with HMB. Moreover, some of the identified miRNAs showed the opposite trend of expression change to this mentioned above (miR-1↓, miR-133↓, miR-206↓), promoting cell differentiation and proliferation in case of miRNA upregulation and downregulation, respectively. They were represented by miR-204 which was upregulated in differentiated human cardiomyocyte progenitor cells [32] and miR-155 (↑) and miR-193a (↑), known to regulate cell differentiation in muscle cells [33] and brown fat cells [34], respectively. All of them possessed the same expression trend which was observed in our experiment in the case of HMB-treated group.

The search of DET for the aforementioned miRNAs was done using Pathway Studio Web and has revealed a large group of genes involved in proliferation and differentiation, the processes previously described to be HMB modulated. The following cell proliferation-related genes were identified: *jak2* (target of identified miR-101, miR-155), *rarg* (miR-142-3p, miR-30c), *pten* (miR-146a, miR-374b, miR-193a), *ets1* (miR-221/222), and *rarb* (miR-146a, miR-146b); cell differentiation-related target genes: *jak2* (miR-155), *pten* (miR-1), *klf4* (miR-1, miR-146a, miR-206) and *ets1* (miR-221/222). Moreover, we identified several target genes which are involved in muscle organ development: *sgcd* (miR-142-3p), *scd* (miR-1, miR-128), *cav3* (miR-101), *tcf12* (miR-101, miR-142-3p, miR-155, miR-204, miR-208, miR-221/222), and *col19a1* (miR-1, miR-206), as modulated in ESC treated with HMB. Special attention deserves miR-206 together with described above miR-1 and miR-133 which regulate expression of one of its potential target genes *cx43* involved not only in muscle development but also in muscle regeneration where its upregulation was observed [35]. The same expression trend of *cx43* was observed in our experiment in HMB-treated group. MiR-206 decreased expression in our experiment may be related to the fact that inhibition of miR-206 robustly increases myotube development [36].

Taken together, changes in expression of pro-proliferative (miR-133a/b, miR-146a/b, miR-222/221) and differentiation-related miRNAs (miR-1, miR-133a/b, miR-155, miR-193a, miR-204, miR-206, miR-221/222, miR-331, miR-324, miR-374, miR-675) were observed following HMB incubation and exposition of ESC cultures to H_2_O_2_, with concomitant changes in expression of their corresponding DET. These results, presenting the pro-proliferation and pro-differentiation effects of the aforementioned miRNAs, could be considered as contradictory, but in fact, both processes are important for proper myogenesis—satellite cell proliferation necessary for proper myofiber regeneration manifested by myoblast fusion with damaged fibers or new myofiber formation, here shown at the very early stage of this process.

### HMB involvement in oxidative stress and inflammation

In our study, we also observed HMB-related changes in the expression of miRNAs playing an important role in modulation of inflammation and oxidative stress. The acute inflammatory response is protective and stimulates repairing of injured tissue [11, 12]. The inflammatory infiltrate is a component of the satellite cell niche and also a source of locally released cytokines which regulate muscle regeneration.

One of the most interesting miRNAs involved in oxidative stress and inflammation appears to be miR-146 family which members are known as negative regulators of inflammatory cytokine expression during immune response [37, 38]. Curtale et al. [39] showed that miR-146b may mediate anti-inflammatory activities and modulates the TLR4 signaling pathway by direct targeting several genes which are most likely targets for our identified miRNAs (*cxcl10*, *tlr4*). Their study also provides evidence for a link between miR-146b and IL-10, indicating that miR-146b induction depends on the activity of IL-10, which is suspected to be realized by muscle cells, both in vivo and in vitro [40]. We did not observe changes in *il-10* mRNA expression in our experiment; however, other interleukin and cytokine gene expression was changed (e.g., *il-5*, *il-6*, *il-13*, *il-15*, *il-18*, *cxcl10*, and *ccl11*).

In muscles, inflammatory response coincides with repair, regeneration, and growth, which involve activation and proliferation of satellite cells, followed by their terminal differentiation. Until now, limited number of data is available to distinguish features of muscle inflammation that promote injury from those that promote growth or repair of muscle. Moreover, dietary supplementation is known to be one of the ways to reduce skeletal and cardiac muscle damage by decreasing the inflammatory and oxidative stress response to exercise in sport horses [41]. In muscles, anti-inflammatory substances (e.g., NSAIDs) are used to control excess local tissue damage by limiting proteolysis from infiltrating inflammatory cells [42, 43]. HMB has been suggested to inhibit inflammation [44]. However, its anti-inflammatory mechanism is still not fully understood. Recent study conducted by Yakabe et al. [44] suggested that HMB has anti-inflammatory potential by downregulation of IL-6 expression. Surprisingly, *il-6* was upregulated in our experiment (FC = 20.01). Interestingly, the local production of IL-6 by skeletal muscle cells and stromal cells promotes activation of satellite cells, thereby increasing myotube regeneration [45]. It is known that IL-6 mediates many aspects of the exercise-induced acute-phase response, including the upregulation of antioxidant defenses as response to oxidative stress [46]. Similar to the aforementioned authors who demonstrated that miR-146b may inhibit pro-inflammatory cytokine secretion, we observed over-expression of both miR-146a and miR-146b. Moreover, miR-155, known to be an immunomodulatory miRNA, acts as a broad limiter of pro-inflammatory gene expression in muscle [47] possessing the same trend which was observed in our experiment in the case of HMB-treated group. This, in turn, suggests that HMB may play an important role in the inflammation processes as an anti-inflammatory factor which could be related to the inhibition of pro-inflammatory cytokine secretion by HMB-induced miR-146 over-expression and activate innate immunity response by miR-155 over-expression.

Interestingly, among DEG (several of them were classified as DET), a large group with the highest FC was involved in different kind of processes related to immunity-acute phase of inflammatory, activation of immunity cells, innate immunity, and pro-inflammatory activity (Table 5). Several of them (e.g., *ccl11*, *ccl2*, *cxcl10*, and *saa1*) were strongly upregulated, and this tendency was previously described as associated with pro-inflammatory activity status [48] which is not fully consistent with tendency of identified in our experiment miRNA expression. Moreover, that a large group of DEG is involved in the inflammatory response and innate immune response in different kind of tissues (*tlr3*, *tlr4*, *tlr10*, *cd40*, *cd44*, *igf2*, *itgb6*, *il-5*, *il-6*, *il-15*, *il*-*23*, and a group of chemokine ligand: *ccl-1*, *ccl-2*, *ccl-5*, *ccl-8*). Both processes are necessary during regeneration, upon injury when immune cells rapidly infiltrate the muscle tissue to remove injured necrotic cells and secrete factors that are essential to activate satellite cells.

**Table 5:** Selected differentially expressed genes (DEG, with the highest FC) and biological processes which they were involved in

No.	Gene symbol	Regulation	Fold change	Description	Accession number	Related processes
1	*cxcl2*	Up	76.31	*Equus caballus* chemokine (C–X–C motif) ligand 2 (CXCL2)	[ENSECAT00000010991]	Expressed in regenerating muscle
2	*cxcl10*	Up	52.29	*Equus caballus* chemokine (C–X–C motif) ligand 10	[NM_001114940]	Acute inflammation Acute skeletal muscle injury
3	*il-6*	Up	20.00	*Equus caballus* interleukin 6 (interferon, beta 2) (IL6)	[NM_001082496]	Mediates the generation and elimination of ROS, stimulates SCs proliferation and hypertrophy
4	*ccl2*	Up	19.41	*Equus caballus* chemokine (C–C motif) ligand 2	[NM_001081931]	Acute inflammation Acute skeletal muscle injury
5	*tlr3*	Up	17.95	*Equus caballus* Toll-like receptor 3 (TLR3)	[NM_001081798]	Oxidative stress, enhances cell viability
6	*saa1*	Up	16.80	*Equus caballus* serum amyloid A1	[NM_001163892]	Acute phase response in skeletal muscle
7	*ccl5*	Up	11.74	*Equus caballus* chemokine (C–C motif) ligand 5 (CCL5)	[NM_001081863]	MAPK cascade, chronic inflammatory response
8	*ccl11*	Up	11.66	*Equus caballus* chemokine (C–C motif) ligand 11 (CCL11)	[NM_001081871]	Smooth muscle cell migration, chronic inflammatory response
9	*sod2*	Up	9.38	*Equus caballus* superoxide dismutase 2	[NM_001082517]	Oxidative stress
10	*xaf1*	Up	7.80	XIAP-associated factor 1	[ENSECAT00000026936]	Apoptosis
11	*parp14*	Up	7.20	Poly(ADP-ribose) polymerase family member 14	[ENSECAT00000026964]	Muscle metabolism
12	*gsdmd*	Up	6.31	Gasdermin d	[ENSECAT00000015748]	Necrosis in inflammation, cell proliferation, induce apoptosis
13	*fgf7*	Up	5.83	*Equus caballus* fibroblast growth factor 7 (FGF7)	[NM_001163883]	Participate in the proliferation of activated satellite cells via the Ras–Raf–MEK–ERK signaling pathway
14	*jak2*	Up	5.14	Janus kinase 2	[ENSECAT00000010434]	Response to injury
15	*cd40*	Up	5.05	*Equus caballus* CD40 molecule, TNF receptor superfamily member 5 (CD40)	[NM_001081902]	Humoral and cell-mediated immune responses, effective immune activation
16	*casp7*	Up	4.93	Caspase 7	[JL623025]	Muscle cell proliferation/survival
17	*psmb9*	Up	4.81	Proteasome (prosome, macropain) subunit, beta type, 9	[ENSECAT00000017937]	Muscle cell differentiation
18	*aox1*	Up	4.10	*Equus caballus* aldehyde oxidase 1	[NM_001287267]	Muscle cell differentiation

The search of target genes for the identified aforementioned miRNAs has revealed a number of genes involved in innate immunity and processes accompanying inflammation, which HMB affects, represented by *jak2* (miR-101, miR-155), *tlr4* (miR-146a/b, miR-155), *cxcl10*, c*xcl11* (miR-146a/b), and *cd47-*target for oxidative stress and inflammation-related miRNAs (miR-221/222 and miR-155). HMB impact on the inflammatory processes and oxidative stress is not fully understood. However, our results show that this substance can modulate in the opposite way expression of pro- and anti-inflammatory miRNAs and genes. We assume this could be linked not only to the potential anti-inflammatory effect of HMB but also to the activation of early (innate) immune response (associated with H_2_O_2_-related damage), which is the initial phase of the regeneration process.

### MicroRNAs related to cell reaction to injury—potential role of HMB as myoprotectant

Among all identified miRNAs, several were known to be involved in cell reaction to injury and different phases of regeneration.

MiR-675 seems to be one of the most interesting miRNAs which is the second of the most downregulated in HMB-treated group and is closely related to regeneration processes. Previous studies showed that miR-675 is expressed in skeletal muscles during myoblast differentiation and muscle regeneration [29]. Another miRNA, miR-146, is related to satellite cell activation [49], myoblast differentiation, and muscle regeneration in vivo [50]. Moreover, also, miR-208 is known to be involved in injury-induced satellite cell activation [51]. We suspect that HMB may stimulate and/or accelerate activation of equine satellite cells at very early stage of the regeneration process. These observations may suggest that HMB acting by the aforementioned miRNA induction may be involved in the satellite cell activation which accompanies regeneration. Hypertrophy is also an important phenomenon of regeneration process in muscle; however, it is related to the final stage of regeneration [13].

We identified several miRNAs that were previously described in relation to muscle hypertrophy. They were represented by downregulation in skeletal muscle hypertrophy miR-1 and miR-133a/b [52, 53] which possessed the same trend as was noticed in our experiment in the case of HMB-treated differentiating ESC cultures. Similar observation was done for miR-142 (↓), which as mentioned above presented one of the highest fold changes (FC = 105.23) among the identified miRNAs, and its downregulation was described during cardiac hypertrophy and is able to inhibit cytokine signaling and function in the myocardium [53]. It is possible that HMB incubation changes the expression of the aforementioned miRNAs facilitating for more efficient late regeneration (not observed during the first day after injury). More research is needed to evaluate the role of miR-133a/miR-1/miR-142-dependent hypertrophy mechanisms of HMB action in the activation of skeletal hypertrophy and muscle regeneration in different physiological and pathological conditions.

Several potential target genes for identified miRNAs were classified as follow: hypertrophy: *tpm3* (miR-1, miR-206), *tpm1* (miR-142-5p), *sgcd* (miR-142-5p), *cacnb1* (miR-208b), *itgav* (miR-142-5p), *itga2* (miR-30c, miR-128); muscle regeneration-related processes and pathways: *cx43* (miR-206), *klf4* (miR-30*c*), and *vegfc* (miR-128, miR-133)*.*

### Other HMB-related miRNAs and genes

The effect of several identified in our experiment miRNAs in muscle tissue is still not clear. However, our results suggest that HMB modulates miRNA expression which was previously described in connection with various physiological and pathological conditions and could also be affected by HMB in injured muscle tissue: miR-374b—related to lipid and protein metabolism [54], miR-14—regulator of mitochondria function in C2C12 [55, 56], miR-532-3p—regulates mitochondrial fission in cardiac muscle [57], miR-30c—cellular adipogenesis and cardiac hypertrophy and ischemia [58], miR-450a/c and miR-142—negative regulators of cardiac hypertrophy [59], and miR-542 family—regeneration of various tissues [60]. Interestingly, several identified genes (among them potential target genes for the aforementioned miRNAs) are also related to amino acid metabolism and protein ubiquitination and proteasome pathways (*rnf6*, *cand2*, *ube2b*, *rnf138*, *rnf19b*, *rnf38*, *ube2l6*, *birc3*, *rnf114*, *mib1*, *trim36*, and *rnf4*) which are closely related to the protein degradation during muscle atrophy in cachexia, and HMB is known to inhibit this process in humans and animals [2, 5, 6].

### Target gene-related signaling pathways

Furthermore, ontological analysis of miRNA DET revealed that a large group of genes was associated with biological processes and signaling pathways that are closely related to the muscle cell, muscle tissue development, and protein metabolism presented in Tables 6 and 7, respectively.

**Table 6:** Selected biological processes identified in DAVID for differentially expressed target genes (DET)

No.	Biological processes	Number of identified genes	List of genes	FDR
1	Muscle organ development	17	*six1*, *tiparp*, *acta1*, *cav3*, *cxcl10*, *col19a1*, *cxadr*, *foxp1*, *fxr1*, *mapk14*, *rarb*, *sgcd*, *zfand5*, *tcf12*, *tagln2*, *tpm1*, *zfpm2*	8.3E−03
2	Modification-dependent protein catabolic process	27	*ube2g1*, *rnf217*, *edem3*, *edem1*, *otub2*, *cul3*, *fbxl19*, *rnf125*, *cyld*, *map1lc3b*, *rnf11*, *hectd1*, *ubr3*, *ube2h*, *mid1*, *hltf*, *trim63*, *march3*, *fem1c*, *dcun1d1*, *nedd4*, *rnf2*, *trim32*, *usp47*, *rnf138*, *cul4b*, *usp25*	1.4E−01
3	Muscle cell differentiation	11	*tbx18*, *acta1*, *cav3*, *cxadr*, *foxp1*,*neo1*, *ntf3*, *rarb*, *sort1*, *syne1*, *tpm1*	2.5E−01
4	Proteolysis involved in cellular catabolic process	27	*ube2g1*, *rnf217*, *edem3*, *edem1*, *otub2*, *cul3*, *fbxl19*, *rnf125*, *cyld*, *map1lc3b*, *rnf11*, *hectd1*, *ubr3*, *ube2h*, *mid1*, *hltf*, *trim63*, *march3*, *fem1c*, *dcun1d1*, *nedd4*, *rnf2*, *trim32*, *usp47*, *rnf138*, *cul4b*,	2.8E−01
5	Muscle tissue development	11	*six1*, *tpm1*, *tiparp*, *acta1*, *col19a1*,*cxadrp2*, *foxp1*, *mapk14*, *rarb*, *zfand5*, *zfpm2*	3.3E−01
6	Protein catabolic process	27	*ube2g1*, *rnf217*, *edem3*, *edem1*, *otub2*, *cul3*, *fbxl19*, *rnf125*, *cyld*, *map1lc3b*, *rnf11*, *hectd1*, *ubr3*, *ube2h*, *mid1*, *hltf*, *trim63*, *march3*, *fem1c*, *dcun1d1*, *nedd4*, *rnf2*, *trim32*, *usp47*, *rnf138*, *cul4b*, *usp25*	4.8E−01
7	Proteolysis	37	*ube2g1*, *rnf217*, *edem3*, *otub2*, *edem1*, *rnf125*, *cul3*, *fbxl19*, *cyld*, *ece1*, *map1lc3b*, *rnf11*, *dpp8*, *nsf*, *hectd1*, *capn6*, *nrip3*, *capn7*, *mmp19*, *ubr3*, *ube2h*, *mid1*, *hltf*, *trim63*, *march3*, *fem1c*, *dcun1d1*, *nedd4*, *rnf2*, *trim32*, *usp47*, *rnf138*, *tep1*, *nln*, *cul4b*, *usp25*, *adamts3*	1.6E−00
8	Regulation of apoptosis	29	*ier3*, *cadm1*, *mcl1*, *tlr4*, *sgms1*, *mcf2l*, *slc11a2*, *cul3*, *map3k7*, *bdnf*, *tiam2*, *sos2*, *ngfrap1*, *faim*, *rarb*, *ip6k2*, *fgd4*, *traf3*, *rarg*, *ntf3*, *arhgef12*, *pla2g4a*, *eya1*, *tnfsf13b*, *dusp1*, *ets1*, *igf2r*, *sort1*, *jak2*	4.5E−00
9	Positive regulation of cellular biosynthetic process	25	*tlr4*, *abca1*, *rarb*, *fam129a*, *znf423*, *rarg*, *epas1*, *ntf3*, *npr1*, *itga2*, *creb5*, *arntl*, *mecom*, *wwtr1*, *pla2g4a*, *ncoa1*, *sp1*, *ets1*, *mapk14*, *six1*, *pdgfra*, *jak2*, *zfpm2*, *smarca1*, *klf4*	8.3E−00
10	Positive regulation of cell proliferation	16	*nampt*, *fgf7*, *itga2*, *vash2*, *foxp1*, *cxcl10*, *cul3*, *cd47*, *vegfc*, *pla2g4a*, *tnfsf13b*, *pdgfra*, *jak2*, *mab21l1*, *tbx18*, *pggt1b*	2.7E−01
11	Positive regulation of inflammatory response	4	*jak2*, *itga2*, *pla2g4a*, *tlr4*	3.1E−01

**Table 7:** Selected pathways identified in DAVID for differentially expressed target genes (DET) and their corresponding miRNAs

No.	Pathway	Pathway *P* value	Number of genes	Gene name	Fold change	FDR	DET-related identified miRNA
1	RIG-I-like receptor signalling pathway	5.2E−04	8	*ddx3x*	1.59 ↑	1.15E−2	miR-101 ↑, **miR-1↓**, **miR-133a/b↓**, **miR-208↓**
				*traf3*	1.31 ↑	1.11E−2	**miR-30↓**, miR-155↑, **miR-133a/b↓**
				*cxcl10*	56.62 ↑	2.16E−5	**miR-142-3p↓**
				*cyld*	1.78 ↑	1.19E−3	**miR-30↓**, **miR-133a/b↓**
				*ikbke*	2.27 ↑	2.44E−3	miR-155↑, **miR-128↓**
				*mapk14*	1.68 ↑	5.43E−3	**miR-128↓**, miR-101↑
				*map3k7*	− 1.58 ↓	4.02E−3	miR-30↓, **miR-101↑**, **miR-155↑**, **miR-204↑**
				*rnf125*	1.88 ↑	9.59E−3	miR-101↑, **miR-142-3p↓**
2	Hyper-trophy cardiomyopathy	1.5E−03	8	*cacnb1*	− 1.42 ↓	1.10E−3	**miR-204↑**, miR-208↓
				*dag1*	− 1.91 ↓	4.43E−3	miR-142-3p↓, **miR-101↑**, miR-30↓
				*itga2*	− 1.32 ↓	7.08E−3	miR-30↓, **miR-101↑**, miR-128↓
				*itgav*	− 1.49 ↓	6.71E−3	miR-142-3p↓
				*prkaa2*	− 1.69 ↓	5.03E−3	miR-30↓, **miR-146a/b↑**
				*sgcd*	1.33 ↑	1.34E−3	**miR-142-3p↓**
				*tpm1*	− 1.85 ↓	2.02E−4	miR-142-3p↓, **miR-542↓**
				*tpm3*	− 2.87 ↓	1.08E−2	**miR-204↑**, **miR-221/222↑**
3	MAPK signalling pathway	7.3E−03	13	*bdnf*	− 2.08 ↓	9.14E−4	miR-1↓, miR-206↓, **miR-155↑**, miR-30↓, **miR-204↑**
				*cacnb1*	− 1.42 ↓	1.10E−3	**miR-204↑**, miR-208↓
				*dusp1*	1.77 ↑	2.80E−3	**miR-133a/b↓**, miR-101↑
				*mecom*	1.50 ↑	1.34E−3	**miR-1↓**, **miR-133a/b↓**, **miR-206↓**, **miR-142-3p↓**
				*fgf11*	− 1.36 ↓	1.11E−2	miR-331↓
				*fgf7*	5.83 ↑	2.33E−3	miR-101↑, miR-155↑, **miR-142-3p↓**
				*mapk14*	1.68 ↑	5.43E−3	**miR-128↓**, miR-101↑
				*map3k7*	− 1.58 ↓	4.02E−3	miR-30↓, **miR-101↑**, **miR-155↑**, **miR-204↑**
				*map3k4*	− 1.36 ↓	9.06E−3	**miR-101↑**, miR-128↓
				*ntf3*	1.72 ↑	2.44E−3	miR-221/222↑
				*pla2g4a*	2.27 ↑	1.39E−3	**miR-142-3p↓**
				*dgfra*	2.82 ↑	1.47E−3	**miR-142-3p↓**
				*sos2*	− 1.62 ↓	9.09E−3	miR-208↓, **miR-204↑**, **miR-221/222↑**
4	p38 MAPK	9.9E−02	4	*mapk14*	1.68 ↑	5.43E−3	**miR-128↓**, miR-101↑
				*map3k4*	− 1.36 ↓	9.01E−3	**miR-101↑**, miR-128↓
				*map3k7*	−1.58 ↓	4.02E−3	miR-30↓, **miR-101↑**, **miR-155↑**, **miR-204↑**
				*dusp1*	1.77 ↑	2.80E−3	**miR-133a/b↓**, miR-101↑
5	Toll-like receptor signalling pathway	1.6E−02	7	*traf3*	1.31 ↑	1.11E−2	miR-30↓, **miR-155↑**, miR-133a/b↓
				*cxcl10*	56.62 ↑	2.16E−5	**miR-142-3p↓**
				*cxcl11*	14.20 ↑	9.51E−5	**miR-1**↓, **miR-206↓**
				*tlr4*	1.44 ↑	9.52E−4	**miR-542↓**, miR-374↑
				*ikbke*	2.27 ↑	2.44E−3	miR-155↑, **miR-128↓**
				*mapk14*	1.68 ↑	5.43E−3	**miR-128↓**, miR-101↑
				*map3k7*	− 1.58 ↓	4.02E−3	miR-30↓, **miR-101↑**, **miR-155↑**, **miR-204↑**
6	Ubiquitin-mediated proteolysis	2.0E−02	8	*rhobtb2*	− 1.62 ↓	9.15E−3	**miR-204↑**, miR-133↓
				*cul3*	− 1.71 ↓	4.76E−5	**miR-101↑**, **miR-193a↑**
				*cul4b*	1.58 ↑	1.08E−3	miR-133a/b↓, **miR-101↑**
				*mid1*	− 1.40 ↓	1.01E−2	miR-374↓, miR-542-3p↓
				*nedd4*	− 1.30 ↓	1.00E−2	miR-30↓, miR-128↓
				*trim32*	1.50 ↑	1.13E−2	miR-155↑, **miR-142-3p↓**
				*ube2g1*	− 1.41 ↓	5.46E−4	**miR-101↑**, miR-30↓,
				*ube2h*	− 1.53 ↓	3.80E−3	miR-1↓, miR-206↓, **miR-204↑**, **miR-101↑**
7	Ras pathway	3.4E−02	6	*tiam2*	− 1.44 ↓	3.27E−3	**miR-101↑**
				*sos2*	− 1.54 ↓	9.09E−3	**miR-193↑**, miR-208↓,
				*ets1*	1.98 ↑	7.19E−3	miR-193↑, miR-208↑, miR-101↑, miR-221/222↑, **miR-1↓**, **miR-206↓**
				*mapk14*	1.68 ↑	5.43E−3	**miR-128↓**, miR-101↑
				*map3k7*	− 1.58 ↓	4.02E−3	miR-30↓, **miR-101↑**, **miR-155↑**, **miR-204↑**
				*map3k4*	− 1.36 ↓	9.01E3	**miR-101↑**, miR-128↓
8	Oxidative stress response	3.9E−02	5	*dusp1*	1.77 ↑	2.80E−3	**miR-133a/b↓**, miR-101↑
				*dusp19*	− 1.55 ↓	2.09E−3	**miR-204↑**
				*mapk14*	1.68 ↑	5.43E−3	**miR-128↓**, miR-101↑
				*map3k4*	− 1.36 ↓	9.01E−3	**miR-101↑**, miR-128↓
				*pla2g4a*	2.27 ↑	1.39E−3	**miR-142-3p↓**

MiRNAs in bold have the opposite expression change to corresponding DET. The arrows indicate the direction of expression change: ↓ and ↑ for down- and upregulation, respectively

Among the most strongly regulated pathways were these previously described as engaged in immunity and inflammation: Toll-like receptor, RIG-I, Ras, MAPK, and ubiquitin-mediated proteolysis. Toll-like receptor pathway is related to the inflammatory activity [61] and different kind of myopathies and could be modulated by miR-155 [62]. RIG-I-like receptor pathway is known to play a crucial role in innate response [63] which is necessary to activate early muscle regeneration and may be modulated by three identified miRNAs: miR-146a, miR-146b, and miR-155. Ras and MAPK pathways promote protein degradation in muscle cells [64] and oxidative stress (closely related to miR-146 activity) [61]. Moreover, MAPK pathway plays a pivotal role in the energy metabolism through modulating lipid metabolism, skeletal muscle growth, and different kind of muscular myopathies and atrophy [65]. Finally, ubiquitin-mediated proteolysis identified as a potential target gene-related pathway is closely related to muscle atrophy by protein degradation.

### Western blot analysis—HMB protective effect on proteins

Western blot analysis was performed to check the level of proteins corresponding with several genes and miRNA. However, the results that were obtained are impossible to use in this way. It is related to the protein degradation which is strongly linked to hydrogen peroxide effect. Previous studies related to hydrogen peroxide effect on different kind of proteins showed that this substance cause protein degradation. For example, exposure of skeletal muscle myotubes to ROS (i.e., hydrogen peroxide) can activate proteases leading to protein degradation as it was described by Li et al. [21] and Clung et al. [55]. Finnegan et al. [9] observed the interactions of a liquid and gaseous H_2_O_2_ with amino acids and proteins (bovine serum albumin and aldolase). In this study, authors observed that two dosages of hydrogen peroxide cause total degradation of BSA (the absence of bands). In our study, we observed similar situation. We checked three different housekeeping genes: β-actin, *gapdh*, and α-tubulin. For all of them, we noticed the same trend of protein degradation in samples where the cells were exposed to hydrogen peroxide (Fig. 6). Moreover, we checked several different proteins where we also noticed protein degradation on a similar level as in samples mentioned above. We suspect that samples where the cells were previously incubated with HMB were protected from protein degradation related to hydrogen peroxide. This degradation has already occurred at the cellular level, not during the sample preparation for western blotting. During the sample preparation procedure, we used RIPA buffer with protease inhibitor cocktail. Based on this observation and previous studies [66–68] related to HMB, we think that this substance may decrease protein degradation in equine satellite cells exposed to hydrogen peroxide; however, the comparison of protein levels between experimental and control conditions is impossible.

### Cell viability, cell damage, and oxidative stress

Five different tests were performed to check the effect of potential antioxidant properties of HMB and its cell protection activity. Based on our results from the test where we observed direct cell oxidative stress together with typical damage (lipid peroxidation together with mitochondria activity changes) related to oxidative stress, it cannot be unambiguously determined whether HMB in experimental dosage has properties that limit oxidative stress (Figs. 8a, b and 9a). However, we observed that HMB increase total antioxidant capacity (Fig. 9b). According to our knowledge, there are limited data considering direct antioxidant activity of HMB; however, as it was mentioned, our study revealed several miRNAs, genes, and pathways related to oxidative stress and antioxidant activity which were modulated by HMB. Increased total antioxidant capacity may represent an adaptive response to the enhanced generation of ROS related to hydrogen peroxide activity or likely responsible for the attenuation of oxidative damage.

Two independent tests showed that HMB increased cell viability: MTT and SYTOX Red Dead Cell (Fig. 7). These observations are consistent with the study presented by Vallejo et al. [69]. Authors also observed that HMB enhanced myoblast viability (in C2C12 cell line). Moreover, earlier study by Pacini et al. [70] showed that HMB significantly prevented dexamethasone-induced cell mortality. Based on our results, we think that HMB may prevents cells from the hydrogen peroxide-induced mortality and enhance ESC’s viability.

## Conclusions

**Fig. 10:**
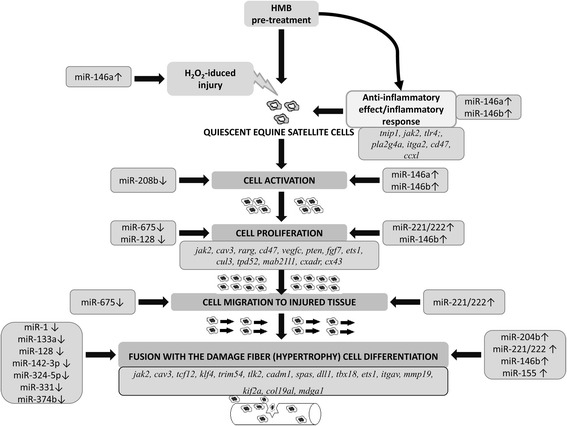
Potential role of HMB-induced miRNAs and selected target genes in muscle regeneration process

Our study presents several new findings of the mechanisms of action of HMB and its potential role in muscle physiology and pathology. We focused on HMB-induced miRNA expression changes previously described as those associated with the muscle tissue injury (inflammation), regeneration, and the accompanying processes such as cell activation, proliferation, migration, and differentiation. The scheme of putative mode of miRNAs and DEG-depended HMB action is proposed in Fig. 10.

We demonstrated for the first time that HMB-treated equine satellite cells exposed to H_2_O_2_ have modulated expression of 27 miRNAs which could affect the abovementioned processes. Many of them were not known for being differentially expressed during myogenic proliferation, differentiation, or processes related to muscle injury and activation in early stage of regeneration. That is why, it would also be interesting to investigate whether some of the abovementioned miRNAs participate in skeletal muscle degeneration/regeneration process as well as degeneration-related equine muscular diseases, such as muscle inflammation related to the extreme effort or recurrent rhabdomyopathy.

Moreover, we found DET for identified HMB-modulated miRNAs which are related to key processes in muscle physiology and pathology. Also, identified pathways MAPK, Ras, and RIG-I together with those involved in oxidative stress response seem to support our knowledge about the potential mechanisms of HMB action. Based on the obtained results, we also believe that HMB increases the survival of cells treated with hydrogen peroxide. Further analyses evaluating the effect of HMB on injured, recovering muscle tissue are needed to verify the collected data.

## Additional files

Additional file 1: **Table S1.** Genes differentially expressed in HMB-incubated equine satellite cells exposed to H_2_O_2_, compared to control. FDR ≤ 0.05, FC ≥ 1.3, *n* = 4. (XLSX 550 kb)

Additional file 2: **Table S2.** Biological function of differentially expressed genes (DEG). (XLSX 28 kb)

## Abbreviations

CTRL: Control condition
DEG: Differentially expressed genes
DET: Differentially expressed target genes
DM: Differentiation medium
DMSO: Dimethylsulfoxide
ESCs: Equine satellite cells
FBS: Fetal bovine serum
GE: Gene expression
GM: Growth medium
HMB: β-Hydroxy-β-methylbutyrate
HS: Horse serum
IB: Incubation buffer
PBS: Phosphate*-*buffered saline
ROS: Reactive oxygen species
